# Negative Effects of Copper Oxide Nanoparticles on Carbon and Nitrogen Cycle Microbial Activities in Contrasting Agricultural Soils and in Presence of Plants

**DOI:** 10.3389/fmicb.2018.03102

**Published:** 2018-12-13

**Authors:** Marie Simonin, Amélie A. M. Cantarel, Armelle Crouzet, Jonathan Gervaix, Jean M. F. Martins, Agnès Richaume

**Affiliations:** ^1^Université de Lyon, Lyon, France; ^2^Université Claude Bernard Lyon 1, Villeurbanne, France; ^3^CNRS, UMR 5557, Microbial Ecology Centre, Université Lyon 1, Villeurbanne, France; ^4^Université Grenoble Alpes, CNRS, IRD, IGE, Grenoble, France

**Keywords:** metal-oxide nanomaterials, agro-ecosystem, microbial ecotoxicology, wheat, nitrification, denitrification, soil respiration, plant-microorganism interactions

## Abstract

Metal-oxide nanoparticles (NPs) such as copper oxide (CuO) NPs offer promising perspectives for the development of novel agro-chemical formulations of pesticides and fertilizers. However, their potential impact on agro-ecosystem functioning still remains to be investigated. Here, we assessed the impact of CuO-NPs (0.1, 1, and 100 mg/kg dry soil) on soil microbial activities involved in the carbon and nitrogen cycles in five contrasting agricultural soils in a microcosm experiment over 90 days. Additionally, in a pot experiment, we evaluated the influence of plant presence on the toxicity of CuO-NPs on soil microbial activities. CuO-NPs caused significant reductions of the three microbial activities measured (denitrification, nitrification, and soil respiration) at 100 mg/kg dry soil, but the low concentrations (0.1 and 1 mg/kg) had limited effects. We observed that denitrification was the most sensitive microbial activity to CuO-NPs in most soil types, while soil respiration and nitrification were mainly impacted in coarse soils with low organic matter content. Additionally, large decreases in heterotrophic microbial activities were observed in soils planted with wheat, even at 1 mg/kg for soil substrate-induced respiration, indicating that plant presence did not mitigate or compensate CuO-NP toxicity for microorganisms. These two experiments show that CuO-NPs can have detrimental effects on microbial activities in soils with contrasting physicochemical properties and previously exposed to various agricultural practices. Moreover, we observed that the negative effects of CuO-NPs increased over time, indicating that short-term studies (hours, days) may underestimate the risks posed by these contaminants in soils.

## Introduction

Copper nanoparticles are increasingly used in various commercial products, including agrochemicals, paints, semiconducting compounds, sensors, catalyzers, and antimicrobial products, which leads to their growing release into terrestrial and aquatic ecosystems (Keller et al., 2017). These emerging contaminants can make their way into soil through direct applications of nanofertilizers or nanopesticides containing copper nanoparticles or through biosolid amendments from wastewater treatment (Lazareva and Keller, 2014; Kah, 2015). Hence, the high reactivity of copper nanomaterials and their established antimicrobial properties raise some concerns about the potential consequences on microbial processes driving soil fertility in agro-ecosystems.

Extensive research has been conducted in the past decade to assess the impact of several metal nanoparticles on soil microbial communities, especially silver and titanium dioxide nanoparticles (reviews by Simonin and Richaume, 2015; McKee and Filser, 2016). However, the effects of copper oxide nanoparticles (CuO-NPs) still remain poorly documented. To our knowledge, only four studies have examined CuO-NP, and these studies were conducted using unrealistic exposure conditions with high concentrations of CuO-NPs ranging from 100 mg/kg to 10 g/kg of soil (Ben-Moshe et al., 2010; Rousk et al., 2012; Frenk et al., 2013; Xu et al., 2015), compared to expected concentrations in soil in the μg/kg to low mg/kg range (Garner and Keller, 2014). Additionally, these studies were performed on one or two model soils that presented predominantly sandy-loam texture. Like any other pollutant, CuO-NP toxicity and bioavailability is likely influenced by soil properties, such as organic matter, pH, texture or ionic strength, and the results obtained in one soil type should thus not be generalized to other soils (Cornelis et al., 2014; Simonin et al., 2015). For example, soil characteristics can influence the transformations of CuO-NPs through processes such as dissolution (Keller et al., 2017). The ionic Cu form can be both highly toxic for soil microorganisms (at high concentrations) and an essential micronutrient for biological growth (at low concentrations, Arguello et al., 2013). Hence, it is hard to predict in which soil types the impact of CuO-NPs would be the most adverse depending on the dissolution rates observed and if CuO-NPs or ionic Cu form would have the larger detrimental effects on soil function. More research needs to be performed to assess the effects of realistic concentrations of CuO-NPs on microbial functioning in soils exhibiting contrasting physicochemical properties.

Moreover, the reliable assessment of the impact of a contaminant on microbial communities in soil needs to consider the influence of plants, especially in the context of an agro-ecosystem where plant-microorganism interactions are intense (Philippot et al., 2013). It is frequently reported that a stressor has no direct effect on microbial communities but that microbial processes are impacted through indirect effects driven by plants in the context of strong plant-soil feedbacks (Cantarel et al., 2015; Simonin et al., 2017; Pommier et al., 2018). For instance, plants can influence the fate and bioavailability of pollutants in soils through soil restructuration by the roots, changes in soil pH, and exudation of organic compounds (Bravin et al., 2012). The presence of plants has also been shown to increase the immobilization and detoxification of Cu in soil (Römkens et al., 1999; Chibuike and Obiora, 2014). A recent study showed that CuO-NPs exhibited slow dissolution rates in soils and that those rates were modulated by the wheat rhizosphere due to direct associations of CuO-NPs to roots, an increase in soil pH and the exudation of small organic acids (Gao et al., 2018). Additionally, the input of nutrient resources through plant exudation can confer a higher resistance and resilience of microbial communities to disturbances (Griffiths and Philippot, 2013). Microbial communities already stressed by low nutrient availability in bulk soils may have less energy available to cope with an external stressor like CuO-NPs in comparison to rhizosphere microbial communities inhabiting nutrient-rich habitats (de Vries and Shade, 2013). Nevertheless, soil ecotoxicological assays on nanomaterials rarely include plants and are mainly performed over short periods of time (2 weeks to 1 month), ignoring the plant-soil feedbacks and indirect effects that can affect pollutant bioavailability and toxicity over long period of times (McKee and Filser, 2016). Specific studies designed to assess how plants modulate NP toxicity for soil microorganisms are clearly needed.

In this study, we performed two experiments to address the following questions: (1) Do CuO-NPs affect microbial function in contrasting soils at relevant low concentrations? (2) Is plant presence influencing the microbial response to CuO-NP exposure? To address the first question, we performed a soil microcosm experiment over 90 days to assess the effects of CuO-NPs at low concentrations (0.1, 1, and 100 mg/kg) on soil microbial activities associated to the carbon and nitrogen (N) cycles (respiration, nitrification, and denitrification) in five contrasting agricultural soils. The effects of CuO-NPs were compared to those of a Cu ion control (CuSO_4_ application) to determine the toxic potential of the Cu nanoparticulate form in comparison to the ionic form. To address the second question, we conducted a follow-up pot experiment to assess the influence of plant presence (winter wheat) on the effects of CuO-NPs on microbial activities over 50 days in the soil that presented the strongest response to the CuO-NPs in the soil microcosm experiment (loam textured LCSA soil).

## Material and Methods

### Soils

For the microcosm experiment, we selected five soils of contrasting textures and exposed to various agricultural practices: a sandy-loam soil used for vegetable production (Brindas), a loam soil under maize-wheat rotations (LCSA), a silty-clay soil under rape-wheat-barley rotations (Commarin), a silty-clay soil used for maize production (Clessé-Maize) and a silty-clay-loam soil from a vineyard (Clessé-Vine). These soils were collected in the Burgundy and Auvergne-Rhône-Alpes Regions (France). More details on the soil characteristics and sample locations are provided in Table 1A. Soils were characterized by the Laboratoire d'Analyse des Sols (LAS, Arras, France) for particle-size distribution (texture class), organic matter content, pH, cation exchange capacity (CEC) and Cu concentration using standardized ISO protocols. We mixed several kilograms of the top soil (0–15 cm) collected at different locations in each field to obtain a representative composite soil sample, and we transported the soils back to the lab in coolers. The soils were then sieved at 2 mm and stored at 4°C for < 1 week before the beginning of the experiment.

**Table 1A:** Main physicochemical characteristics of the five soils used in the microcosm experiment.

**Soils**	**Sampling location**	**Texture**	**Sand (%)**	**Loam (%)**	**Clay (%)**	**CEC (cmol(+)/kg)**	**OM (%)**	**Water holding capacity (%)**	**pH**	**Cu (mg/kg)**
Brindas	Brindas, France (45°43′40.8″N 4°43′35.4″E)	Sandy-Loam	68.4	14.7	16.9	11.5	2.09	20	7	20.1
LCSA	La Côte St André, France (45°22′39.3″N 5°16′06.1″E)	Loam	37.5	42.7	19.8	8.79	2.23	27	6.4	13.2
Commarin	Commarin, France (47°14′37.0″N 4°38′53.1″E)	Silty-Clay	8.2	49.8	42	17.4	4.72	47	6.94	23.2
Clessé-Maize	Clessé, France (46°25′03.5″N 4°47′58.8″E)	Silty-Clay	7.3	48.9	43.8	20.7	2.88	28	8.21	35.5
Clessé-Vine	Clessé, France (46°25′03.1″N 4°48′03.5″E)	Silty-Clay-Loam	12.5	58.6	28.9	14.8	2.59	26	7.75	47.5

### Nanoparticle Characteristics

For the experiments, we used manufactured powdered CuO-NPs commercialized by Sigma-Aldrich. The CuO-NPs had a nominal size < 50 nm and a specific surface area of 23 m2 g^−1^, according to the manufacturer information. The intrinsic primary particle size was verified using a ZEISS Ultra 55 scanning electron microscopy field emission gun (SEM-FEG) and energy dispersive spectroscopy (EDS) with a SDD detector (BRUKERAXS-30 mm^2^). On average, the CuO-NPs measured 57.0 ± 18 nm. The apparent hydrodynamic diameter and zeta potential of the CuO-NPs were characterized using Dynamic Light Scattering (DLS) with a NanoZS (Malvern Instruments, UK, laser of 638 nm wavelength) in 50 mg CuO-NPs L^−1^ of soil solution prepared according to Simonin et al. (2015). All CuO-NP suspensions were dispersed using ultrasonication for 5 min before use to ensure suspension homogeneity. The CuO-NP hydrodynamic diameters and zeta potentials measured in the five soil solutions are presented in Table 1B. The dissolution of CuO-NPs was assessed in triplicate soil solutions spiked with 50 mg L^−1^ CuO-NPs for 1, 7, 30, and 90 days and incubated in the dark at 28°C in plasma flasks (150 mL) under the same conditions as the soil microcosm experiment described below. At each date, we isolated the dissolved Cu fraction in the soil solutions using ultrafiltration tubes (5 KDa) centrifuged for 45 min at 6,000 g and determined the Cu concentration using ICP-OES (Varian 700-ES, Varian Inc. Scientific Instruments, Palo Alto, USA). In the five soil solutions, we observed < 2% cumulative dissolution (Table 1B) of the CuO-NPs during the 90 days of incubation.

**Table 1B:** CuO-NP characterization (at 50 mg/L) in the five soil solutions.

**Soil Solutions**	**Hydrodynamic diameter (nm)**	**Zeta potential (mV)**	**Cumulative CuO-NPs dissolution over 90 days (%)**
Brindas	57.6	−19.1	1.23
LCSA	46.8	−13.8	1.61
Commarin	61.8	−14.3	0.77
Clessé-Maize	101.2	−19.9	0.50
Clessé-Vine	75.6	−21.3	0.83

### Soil Microcosm Experimental Design

In the microcosm experiment, we exposed the soils to five treatments, including three concentrations of CuO-NPs (0.1, 1, and 100 mg/kg), an ionic Cu treatment of copper sulfate (CuSO_4_) at 100 mg/kg and a control treatment that received no Cu addition. The CuO-NP concentrations were selected to cover both a range of low realistic concentrations in the ppb range (0.1 mg/kg, Keller et al., 2017) and a higher concentration representing an accidental spill (100 mg/kg). For each treatment, 600 g (equivalent dry weight) of each soil were spiked with an ultrasonicated CuO-NP or CuSO_4_ solution at a concentration of 0, 1.79, 17.9, or 1,790 mg/L in ultrapure water to achieve the required final concentrations. The CuO-NPs suspensions were added homogeneously to the soils using a multichannel pipette and then soils were thoroughly mixed for 10 min to ensure a uniform exposure. Soil moisture was adjusted to the water holding capacity specific to each soil. 50 g (equivalent dry weight) of each spiked soil were then transferred into 150 mL glass plasma flasks sealed with rubber stoppers to maintain constant soil moisture during the duration of the experiment. The microcosms were incubated in the dark at 28°C for 7 or 90 days and were weekly aerated under a sterile atmosphere for 5 min to renew the atmosphere in the flask. Each treatment was replicated in six independent microcosms, resulting in the incubation and analysis of 300 microcosms (6 replicates × 5 soils × 5 treatments × 2 dates). At the end of each incubation time (7 or 90 days), the microcosms were subsampled for microbial activity measurements (stored at 4°C and analyzed within 3 days), DNA extractions and subsequent qPCR measurements (stored at −20°C). On the remaining soil, on day 7, we measured the soil pH using the ISO 10390 protocol for the different treatments. The different CuO-NP treatments did not induce significant pH changes in the five soils studied (data not shown).

### Pot Experiment With *Triticum aestivum*

Using a pot experiment under greenhouse conditions, we compared microbial responses between unplanted and planted conditions in the LCSA soil (Table 1A) exposed to two concentrations of CuO-NPs (1 and 100 mg/kg) and no Cu addition (control). Five replicates by treatment were used, and all experiments were conducted using winter wheat (*Triticum aestivum*) grown in pots without a N supply. Two seeds previously germinated in a humid chamber for 1 week were sown per pot (12 × 12 × 12 cm) containing 1.5 kg of sieved loam soil (< 2 mm) collected at La Côte Saint-André (LCSA, Table 1A). Plants were grown for 50 days in a climatic chamber (Fitoclima 10,000 EH, ARALAB) with 16 h light-−8 h night; day and night temperatures of 21 and 18°C, respectively; CO_2_ concentration of 350 ppm; and chamber relative air humidity of 70%. Each pot was watered three times a week. After 50 days of incubation, the microbial activities, and plant biomass were determined. The leaf and root systems of planted pots were dried at 105°C for 2 days to measure above-ground, below-ground and total plant dry masses (g dry weight) and to assess whole plant shoot/root ratio. pH was determined for each soil sample as explained in section Soils.

### Microbial Activity Measurements

#### Substrate-Induced Respiration (SIR)

To measure substrate-induced respiration (SIR) at each time point, 10 g (equivalent dry weight) of fresh soil was placed in a new glass plasma flask to which we added 0.5 mL of a glucose solution (1.2 mg C-glucose g^−1^ dry soil) as a non-limiting carbon source for microbial respiration (Patra et al., 2005). The flasks were hermetically sealed with a rubber stopper and incubated at 28°C for 7 h. CO_2_ accumulation in the flask was measured every hour using a gas chromatograph (Micro GC R3000, SRA Instrument, Marcy L'Etoile, France).

#### Nitrification Enzyme Activity (NEA)

To measure nitrification enzyme activity (NEA), 3 g (equivalent dry weight) of fresh soil was incubated with 6 ml of (NH_4_)_2_SO_4_ solution (50 μg N-N${\text{H}}_{4}^{+}$ g^−1^ dry soil) in a new plasma flask (Dassonville et al., 2011). Distilled water was added to each sample to achieve 24 ml of total liquid volume in flasks. The flasks were sealed with Parafilm® and incubated at 28°C under constant shaking (140 rpm). 1.5 ml of soil slurry were sampled after 2, 4, 6, 8, and 10 h of incubation, filtered at 0.2 μm and stored in vials at −20°C until measurement of ${\text{NO}}_{2}^{-}$ and ${\text{NO}}_{3}^{-}$ concentrations using an ion chromatograph (ICS 900, Dionex, Salt Lake City, USA).

#### Denitrification Enzyme Activity (DEA)

To measure denitrification enzyme activity (DEA), 10 g (equivalent dry weight) of fresh soil was placed in a new plasma flask hermetically sealed with a rubber stopper (Bardon et al., 2014). The atmosphere of the flasks was replaced by a 90% helium and 10% acetylene mixture to obtain anaerobic conditions and inhibit the nitrous oxide reductase that catalyzes the final step of denitrification converting N_2_O in N_2_. Distilled water (1 ml) containing KNO_3_ (50 μg ${\text{N-NO}}_{3}^{-}$ g^−1^ dry soil), glucose (500 μg C-glucose g^−1^ dry soil), and glutamic acid (500 μg C-glutamic acid g^−1^ dry soil) was added through the rubber stopper using a syringe to ensure non-limiting amounts of carbon and ${\text{NO}}_{3}^{-}$ for denitrification activity. The flasks were incubated at 28°C for 8 h. After 2 h, N_2_O concentration in the atmosphere of the flasks was measured every hour using the gas chromatograph described in section Substrate-Induced Respiration (SIR).

### Microbial Abundance Measurements

Soil DNA was extracted from 0.5 g of frozen soil using the FastDNA® Spin Kit for Soil (MPbio, California, USA) following the manufacturer's instructions. DNA quantification was performed using the Qubit® dsDNA BR Assay Kit on a Qubit® 2.0 fluorometer.

The abundance of the total bacterial community, *amoA* nitrifiers (AOA and AOB) and denitrifiers were measured with quantitative PCR using a Lightcycler 480 (Roche Diagnostics, Meylan, France) following the protocols described in Simonin et al. (2015, 2016). For total bacterial abundance, we amplified the *rrs* gene encoding for 16S rRNA using the universal primers 519F and 907R targeting the V4-V5 region. For the nitrifiers, the *amoA* functional gene was amplified using gene primers amoA_1F and amoA_2R for the AOB, and CrenamoA616r and CrenamoA23f for the AOA. Denitrifier abundance was measured with the *nirS* functional gene encoding Cu-containing NO_2_ reductase. Amplification was performed using nirSCd3aF and nirSR3cd gene primers. All reactions were performed in duplicate using 1X QuantiTectSybrGreen PCR Master Mix (Qiagen, Courtaboeuf, France) and serial dilutions of DNA standards for each gene were included (10^2^ to 10^7^ gene copies μL^−1^).

### Statistical Analyses

The results are presented as means (±standard errors). In the pot experiment, microbial activities measured in the planted condition are presented as the percentage relative to the unplanted condition in the same Cu treatment (control, 1 mg/kg CuO-NP or 100 mg/kg CuO-NP). We tested the effects of the CuO-NP and ionic Cu treatments on microbial and plant endpoints for each soil tested using a generalized linear model with the *glm* function (fitted with Gaussian or Gamma probability distribution) at the two sampling dates. We then performed *post-hoc* tests using the lsmeans package in the R software version 2.3.2 (R Core Team, 2015). We used a *P* < 0.05 threshold for significance. Linear regression of microbial activities and abundances were explored and described with the Spearman correlation coefficient.

## Results

### Effect of CuO-NPs on Soil Microbial Activities in Contrasting Soils—Soil Microcosm Experiment

**Figure 1:**
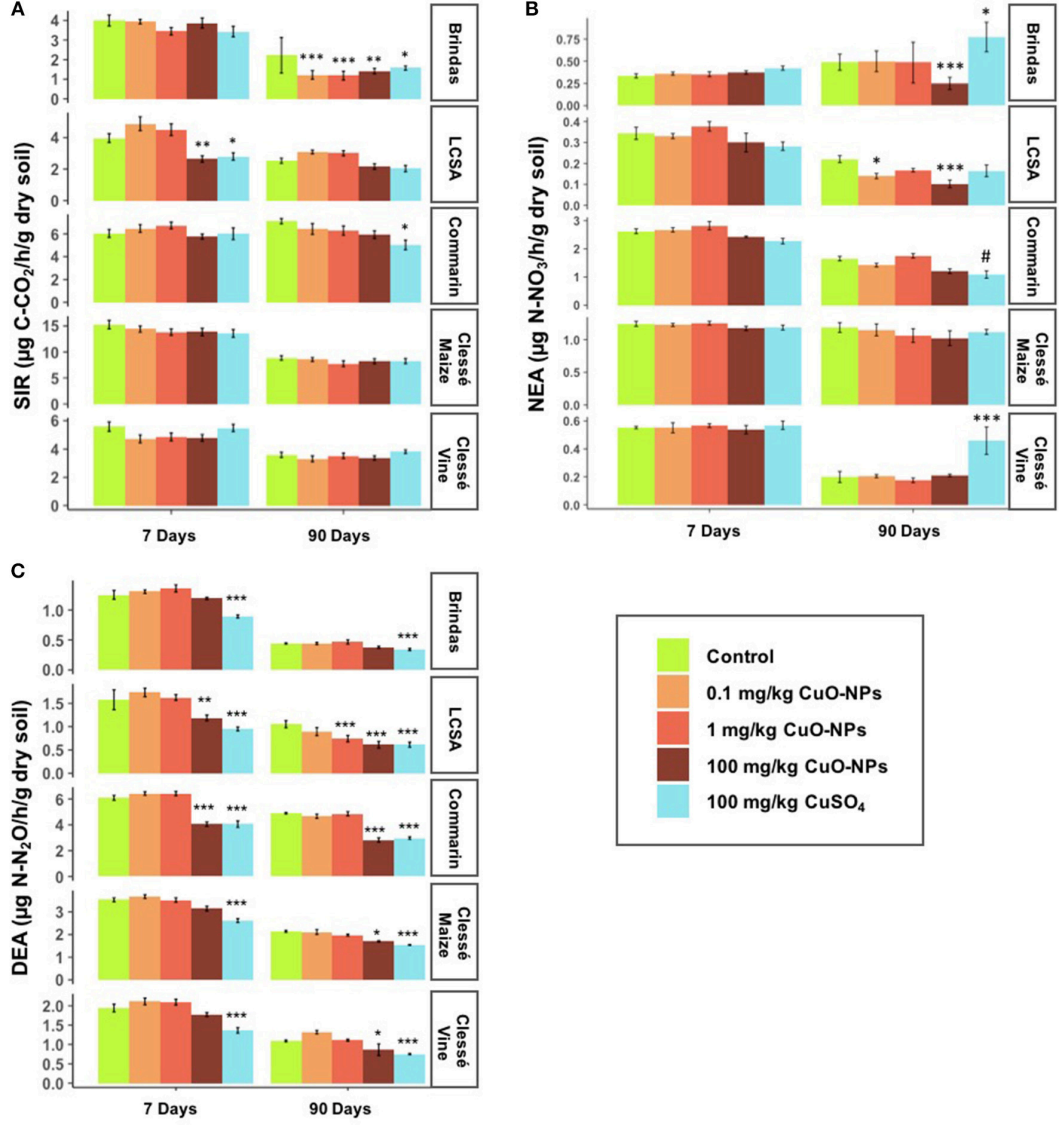
Effect of CuO-NPs and CuSO_4_ on microbial activities in the five soils on days 7 and 90. **(A)** Substrate-induced respiration (SIR), **(B)** Nitrification enzyme activity (NEA), and **(C)** Denitrification enzyme activity (DEA). The symbols represent the significant effects of the treatments compared to the controls: #*P* < 0.06; ^*^P < 0.05; ^**^P < 0.01; ^***^P < 0.001. Note that the scale of the y-axes is different for each soil.

In the microcosm experiment, we observed that the effects of CuO-NPs and CuSO_4_ differed between the five soils (Figure 1). On day 7, none of the treatments altered NEA, regardless of soil type (Figure 1B). SIR significantly decreased only in LCSA soil exposed to 100 mg/kg CuO-NPs (−33%) or CuSO_4_ (−29%). The DEA decreased only in the 100 mg/kg CuSO_4_ treatment in all the soils (Figure 1C, −26 to −40%), while the same concentration of CuO-NPs significantly reduced this activity only in LCSA (−25%) and Commarin soils (−33%). The 0.1 and 1 mg/kg CuO-NPs did not have any effects on DEA on day 7.

On day 90, SIR significantly decreased in all the treatments in Brindas soil (Figure 1A), including the 0.1 and 1 mg/kg CuO-NP doses that led to the highest reductions (−45 and −47%, respectively). In the other soils, SIR activity was not affected except in Commarin soil with 100 mg/kg CuSO_4_ (−29%). NEA in Brindas soil was reduced only by the 100 mg/kg CuO-NP treatment (−49%, Figure 1B), while this activity was decreased in LCSA soil by the 0.1 and 100 mg/kg CuO-NP treatments (−37 and −54%). CuO-NPs did not affect NEA in the three other soils, but CuSO_4_ treatment led to a decrease in NEA in Commarin soil (−34%) and an increase in NEA in Brindas (+58%) and Clessé-vine soils (+131%). On day 90, DEA was still significantly suppressed in the 100 mg/kg CuSO_4_ treatment in all the soils (−23 to −42%, Figure 1C). The same concentration of CuO-NPs caused a significant decrease from −21 to −42% in all the soils except Brindas soil. The 1 mg/kg CuO-NP treatment significantly decreased DEA only in LCSA soil (−30%), but the lowest concentration (0.1 mg/kg) had no effect on this microbial process.

### Effect of CuO-NPs on Microbial Abundance and Correlations With Microbial Activities—Soil Microcosm Experiment

On day 7, the different treatments did not alter the microbial abundance (data not shown) and had only limited effects on day 90 (Figure S1). The AOA abundance decreased with 100 mg/kg CuO-NPs in LCSA soil (Figure S1B, −57%). The same exposure with CuO-NPs and CuSO_4_ in Commarin soil resulted in a similar decrease in the AOB abundance (Figure S1C, −45 and −50%, respectively). The abundance of denitrifiers bearing the *nirS* gene was also reduced in the 1 and 100 mg/kg CuO-NP treatments in this soil (−48 and −45%, Figure S1D).

**Figure 2:**
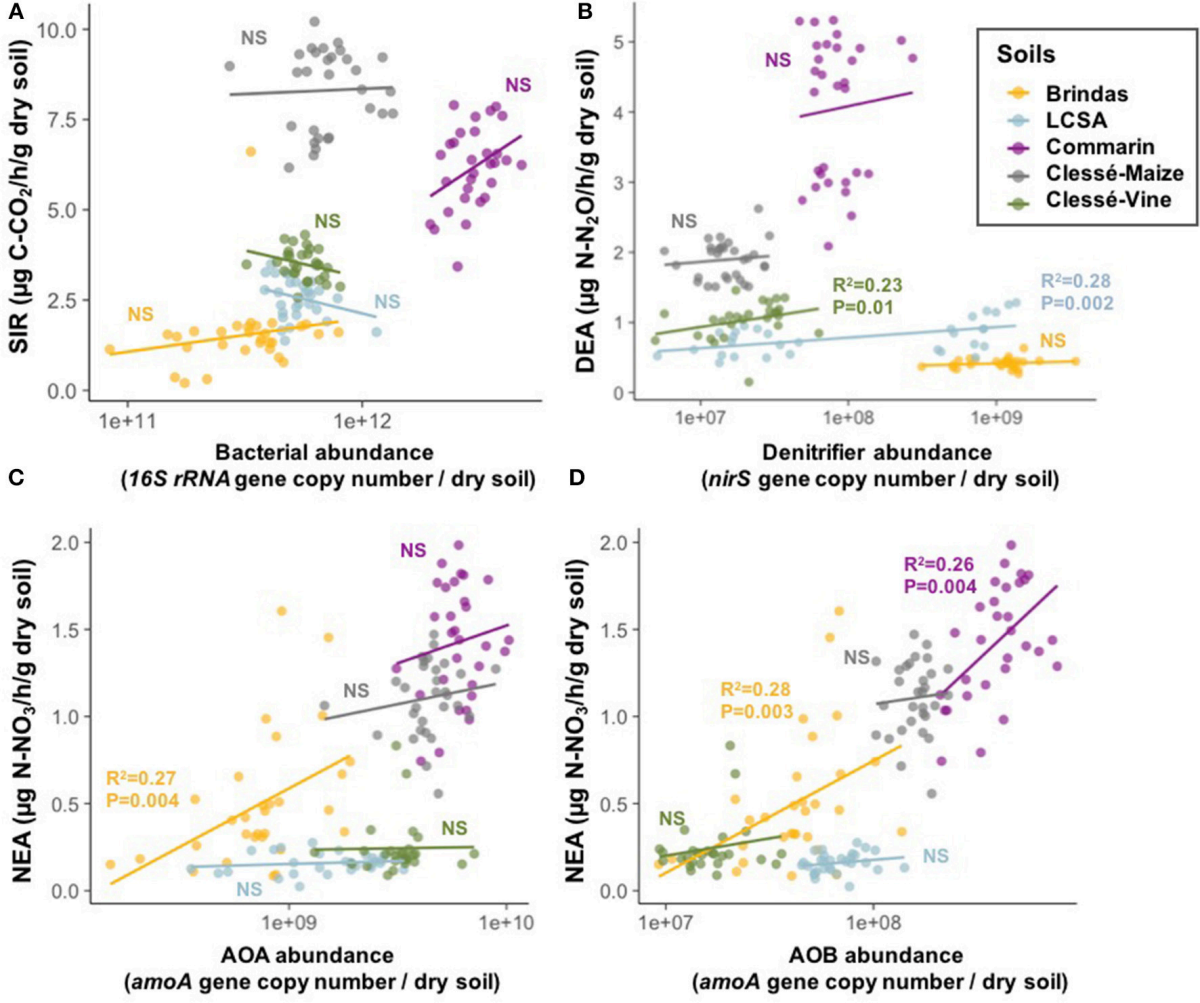
Correlations between microbial activities and microbial abundance in the five soils on day 90. **(A)** SIR vs. bacterial abundance, **(B)** DEA vs. *nirS*-bearing denitrifier abundance, **(C)** NEA vs. AOA abundance and **(D)** NEA vs. AOB abundance. When a correlation is significant, the *R*^2^ and *P*-value are indicated, otherwise NS for Non-Significant is displayed.

We found that SIR was not significantly correlated to bacterial abundance in the five soils studied (Figure 2A). DEA was positively correlated to *nirS*-bearing denitrifier abundance in LCSA and Clessé Vine soils (Figure 2B). NEA was positively correlated to both AOA and AOB abundance in Brindas soil (Figures 2C,D) and to AOB abundance in Commarin soil (Figure 2D). In LCSA, Clessé-Maize and Clessé-Vine soils, NEA was not correlated to nitrifier abundance.

### Influence of Plant Presence on CuO-NP Effects on Soil Microbial Activities in LCSA Soil—Pot Experiment

**Figure 3:**
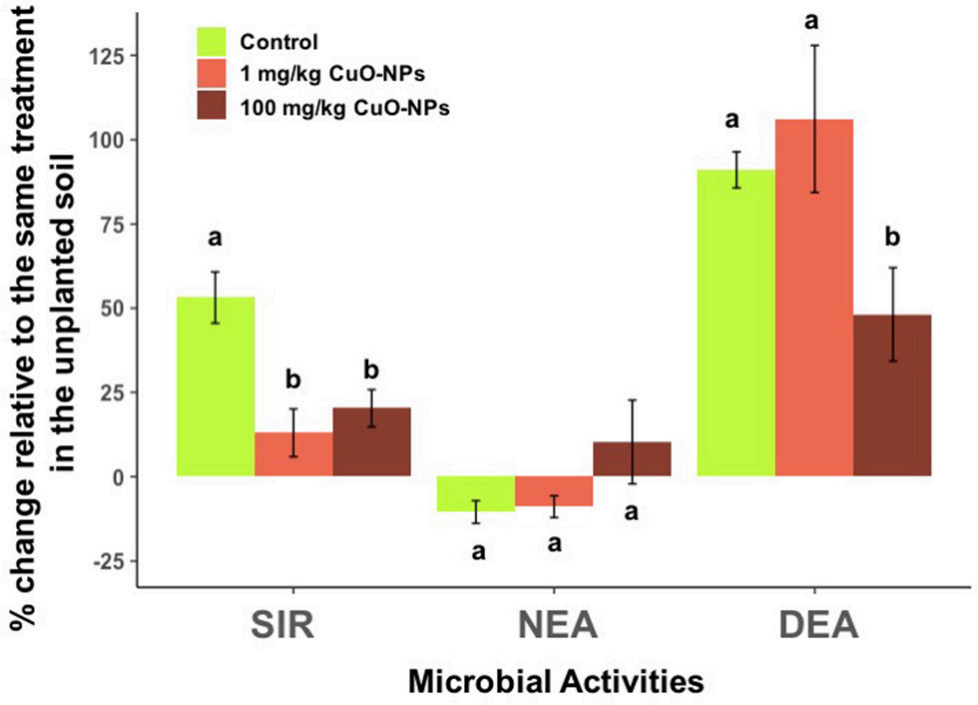
Effects of CuO-NPs on microbial activities expressed as percentage relative to the same treatment in the unplanted soils after 50 days of exposure. Different letters indicate a significant difference between the treatments for a given microbial activity.

By comparing microbial activities in unplanted and planted pots, we observed that plant presence had a positive effect on SIR and DEA [*F*_(1, 1)_ = 27.2, *P* < 0.0001 and F_(1, 1)_ = 81.3, *P* < 0.0001, respectively], but not on NEA after 50 days of exposure (*P* = 0.39; Figure 3). We found that the effects of the CuO-NP treatment could differ significantly between unplanted and planted soils for DEA (significant Copper treatment x Plant presence interaction, *F*_(1, 2)_ = 6.1, *P* = 0.007).

For SIR, the positive effect of plants (average +53%) was negated by the addition of 1 and 100 mg/kg of CuO-NPs (average +13 and +20%, respectively; Figure 3). Similarly, the stimulation of DEA associated with the plant presence was reduced two-fold when the soil was exposed to 100 mg/kg CuO-NPs (average +91% in control and +48% in 100 mg/kg CuO-NPs; Figure 3). However, NEA was not influenced by plant presence nor by the CuO-NP treatments (Figure 3).

### Effects of CuO-NP on Plant Biomass and Soil pH—Pot Experiment

**Figure 4:**
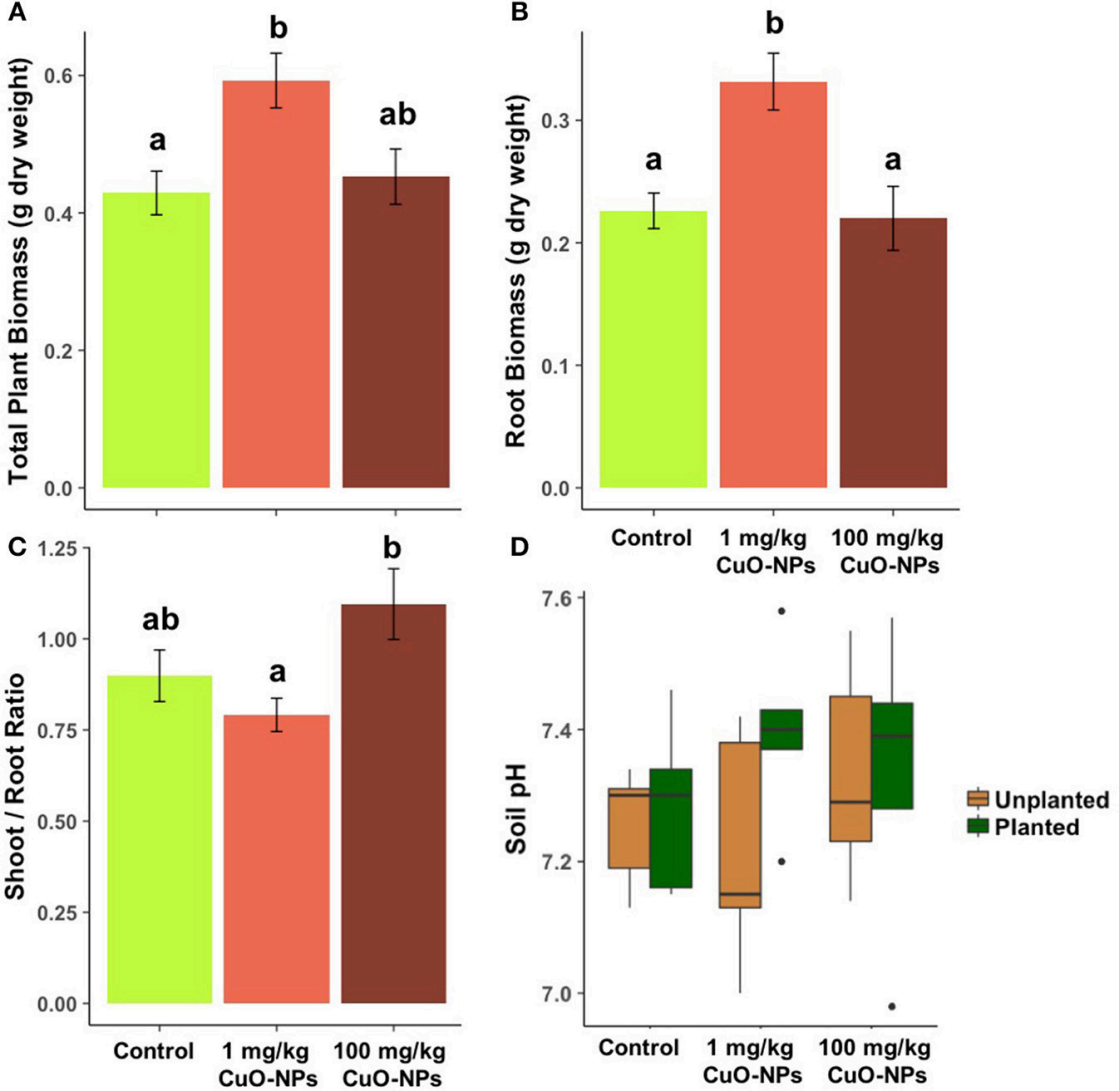
Effects of CuO-NPs on wheat **(A)** total plant biomass, **(B)** root biomass, and **(C)** shoot/root ratio. **(D)** pH in the unplanted and planted soil after 50 days of exposure. Different letters indicate a significant difference between the treatments for a given endpoint.

In the pot experiment conducted with LCSA soil, the CuO-NP treatments significantly affected the growth of wheat after 50 days of exposure (Figure 4). Total and root biomasses significantly increased in the 1 mg/kg CuO-NP dose compared to the control (+38% and +47%, respectively; Figures 4A,B). A significant increase in the shoot/root ratio was observed in the 100 mg/kg CuO-NP treatment compared to the 1 mg/kg CuO-NP treatment (+38%; Figure 4C). No significant effect was observed on the shoot biomass. No effect of CuO-NP treatment or plant presence was observed on soil pH values (Figure 4D).

### Correlations Between Microbial Activities and Plant Biomass or Soil pH—Pot Experiment

**Figure 5:**
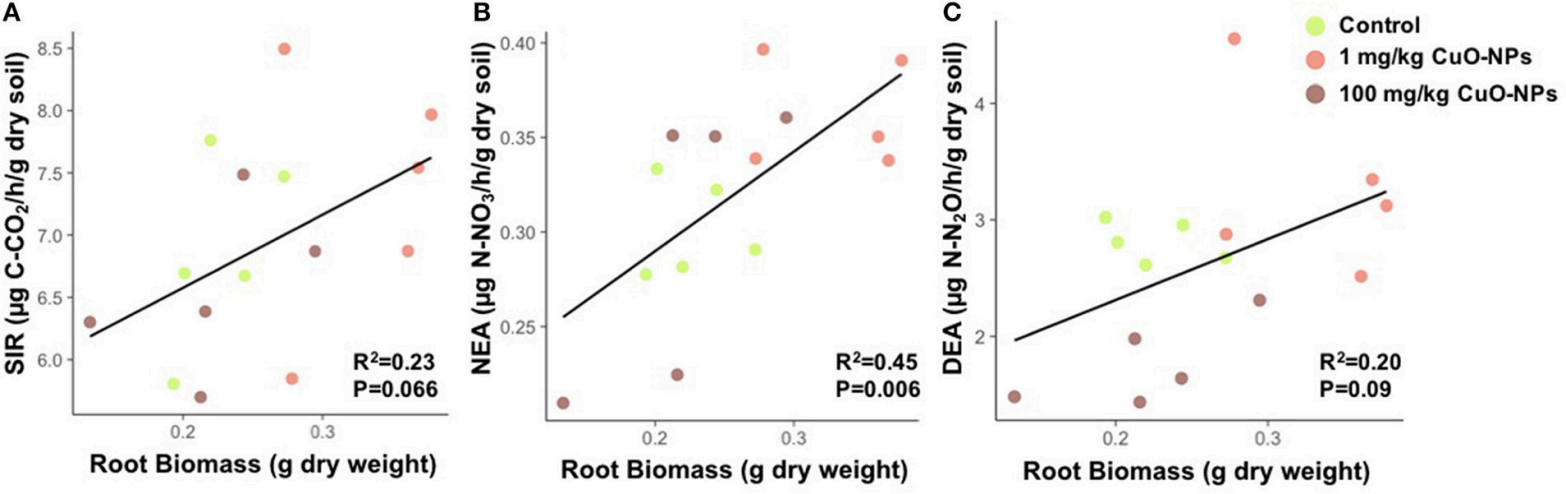
Correlations between microbial activities and root biomass after 50 days of exposure. **(A)** SIR vs. root biomass, **(B)** DEA vs. root biomass, and **(C)** NEA vs. root biomass. The *R*^2^ and *P*-values are indicated.

SIR and NEA were positively correlated to root biomass [*R*2 = 0.23; *F*_(1, 13)_ = 4.1, *P* = 0.066 and *R*2 = 0.45; *F*_(1, 13)_ = 10.7, *P* = 0.006, respectively; Figures 5A,B]. We also observed a positive relationship between DEA and root biomass, but the correlation was only marginally significant [*R*2 = 0.20; *F*_(1, 13)_ = 3.3, *P* = 0.09; Figure 5C]. Further confirming the influence of root biomass on microbial activities, we found that the shoot/root ratio was negatively correlated to microbial activities linked to the N cycle [*R*2 = 0.57; *F*_(1, 13)_ = 17.1, *P* = 0.00012 and *R*2 = 0.48; *F*_(1, 13)_ = 11.9, *P* = 0.004, respectively for NEA and DEA] but not to SIR.

Plants also influenced the relationships between N cycle microbial activities in this experiment. DEA was positively correlated to NEA in the unplanted and planted soil. However, the correlation was stronger in the unplanted soil [*R*2 = 0.49; *F*_(1, 13)_ = 12.6, *P* = 0.0036] than in the planted one [*R*2 = 0.34; *F*_(1, 13)_ = 6.77, *P* = 0.022]. When considering all treatments together, the microbial activities were not significantly correlated to soil pH (SIR: *P* = 0.17; NEA: *P* = 0.84; DEA: *P* = 0.65). A correlation between SIR and pH was only found for the 1 mg/kg CuO-NP dose [*R*2 = 0.42; *F*_(1, 8)_ = 5.7, *P* = 0.043].

## Discussion

### Distinct Effects of CuO-NPs and Ionic Cu on Microbial Activities

On the three microbial activities measured (DEA, NEA, and SIR), ionic Cu contamination (CuSO_4_) led to either (i) similar decreases compared to CuO-NP addition (e.g., DEA on day 90), (ii) higher decreases compared to CuO-NP exposure (e.g., on DEA on day 7) or (iii) increases in microbial activity, while CuO-NP exposure had no effect or caused a decrease (e.g., NEA on day 90). These results provide evidence that the consequences of CuO-NPs and ionic Cu on soil microbial activities are distinct. In particular, CuO-NPs never presented stimulatory effects on the activities measured, while CuSO_4_ addition stimulated NEA in two different soils (Brindas and Clessé Vine) after 90 days. This result might be explained by the Cu requirement of ammonia monooxygenase enzymes catalyzing the first step of NEA (Wagner et al., 2016). Hence, after 90 days, we can hypothesize that the limitation of bioavailable Cu might have been alleviated by CuSO_4_ addition but not by CuO-NP addition in those two soils. Overall, the contrasted effects of the CuO-NPs and ionic Cu treatments on microbial activities are likely explained by the very low dissolution of CuO-NPs in the five soils. The dissolution of CuO-NPs in the five soil solutions was below 2% after 90 days. This result is supported by the study of Gao et al. (2018) that also shows slow dissolution rates of CuO-NPs in an agricultural soil. Altogether, these findings suggest that the effects of CuO-NPs were likely principally driven by the Cu nanoparticulate form and not the ionic Cu form.

### Limited Effects of CuO-NPs on Microbial Activities at Low Concentrations and High Decreases of DEA in Most Soils

The low CuO-NP concentrations tested (0.1 and 1 mg/kg) had no effect on the studied microbial activities, with the exception of the decrease on day 90 of SIR in sandy-loam Brindas soil, and of NEA and DEA in loam LCSA soil. Hence, the results of this microcosm experiment show that CuO-NP exposures at low and relevant concentrations have limited effects on soil microbial activities involved in carbon and N cycles, but that soils presenting a coarse texture (low clay content) might be occasionally affected.

The lowest concentration tested previously in the literature was 100 mg/kg CuO-NPs; this concentration led to decreases in different microbial enzyme activities (urease, dehydrogenase, phosphatase) in a flooded paddy soil (Xu et al., 2015). Similarly, in our study, we observed that 100 mg/kg CuO-NP caused significant reductions in DEA, NEA, and SIR. In particular, DEA was found to be the most sensitive microbial process to CuO-NP exposure in the short-term (7 days) and longer term (90 days). On day 90, four of the five soils exhibited significant DEA decreases ranging from 21 to 42% in the presence of 100 mg/kg CuO-NP. The medium and fine texture LCSA (loam) and Commarin (silty-clay) soils were found to be the most sensitive soils for the DEA activity. SIR and NEA were less affected by CuO-NP exposure, and significant decreases were observed only in the two soils with the coarse and medium textures (Brindas and LCSA). These findings show that CuO-NPs can have detrimental effects at high concentration (100 mg/kg) in agricultural soils exhibiting very contrasting textures, OM content, pH, and agricultural practices.

In contrast to other heavy metal contaminants, we did not observe clear patterns indicating a higher toxicity of CuO-NPs on DEA in soils with a coarse texture (high sand content) and low OM content as usually reported (Giller et al., 1998; Kuan et al., 2006; Zhang et al., 2016). Several studies report that NP toxicity varies strongly depending on the types of soils tested but the observed patterns associated with soil texture, pH, and OM content differ both in function of the NP tested and between studies (McKee and Filser, 2016). More in-depth characterization of the transformations experienced by CuO-NPs in diverse soils and of the soil parameters driving CuO-NP bioavailability and toxicity are needed.

Denitrifiers have often been found to be insensitive to many toxicants and to even increase in relative abundance in polluted sites, while nitrifiers are frequently reported to be very sensitive to metal pollution (Bissett et al., 2013). Therefore, we were surprised to observe that DEA was the most sensitive microbial process to CuO-NPs in our experiment, especially in comparison to NEA. Denitrifiers exhibit a higher diversity and functional redundancy compared to nitrifiers and also have a larger niche breadth (facultative anaerobes, diversity of organic substrates) that make the DEA generally more resistant and resilient to disturbances (Griffiths and Philippot, 2013). Moreover, as nitrification and denitrification are tightly coupled, the decline of DEA is often the consequence of a decrease in NEA. Thus, this study shows that in contrast with other NPs (Simonin et al., 2016), CuO-NPs have more detrimental effects on DEA than on NEA in the short and long-term in different soils.

The effects of CuO-NPs on microbial activities did not seem to be related to strong decreases in the abundance of the different microbial groups driving these processes (total bacteria, AOA, AOB, denitrifiers). The microbial abundances remained mainly unchanged by the treatments, and correlations between microbial activities and abundances were observed only in a few soils. Our results suggest that the DEA inhibitions in Brindas and Clessé-Maize soils were not associated with changes in the abundance of *nirS*-bearing denitrifiers. However, the significant correlations between DEA and denitrifier abundance in the LCSA and Clessé-Vine soils indicate that the decreases in denitrification rates were at least partially related to a decrease in denitrifier abundance. More research would be required to determine whether CuO-NPs can affect enzyme synthesis and functioning or lead to modifications in microbial community structure over time that could result in reductions in key microbial activities like denitrification.

### CuO-NP Effects Increase Over Time

Consistent with many other ecotoxicological studies looking at NP toxicity, we observed that the detrimental effects of CuO-NPs increased over time (Simonin et al., 2015, 2016; McKee and Filser, 2016). In this study, two-third of the significant effects observed on the microbial activities occurred on day 90. These toxic effects detected and/or increasing after longer exposures could be explained by several abiotic factors (e.g., pH, DOC, ionic strength) that are dynamic over time and could transform CuO-NPs into an aged form more bioavailable or toxic for microorganisms (Cornelis et al., 2014). A modification of soil abiotic parameters over the course of the incubation can affect not only CuO-NP fate but also the soil microbial community structure. The temporal variation in microbial community composition and the loss or decline of sensitive taxa to CuO-NPs over time may be a key explanation for the decreases in microbial activities observed after 90 days. Altogether, these results reinforce the idea that short-term ecotoxicological assays may not be adapted to assess the risks associated to NP contamination in soils and may lead to an underestimation of their ecological consequences.

### Stimulation of Microbial Activities by Plant Presence Does Not Mitigate CuO-NP Toxicity

In the pot experiment, we found that plant presence strongly stimulated heterotrophic microbial activity (i.e., SIR and DEA) likely through inputs of carbon in the rhizosphere as suggested by the positive correlations between the microbial activities and root biomass (Smith and Tiedje, 1979; Klemedtsson et al., 1987). However, this stimulatory effect of the wheat did not counteract or dampen the negative effects of CuO-NPs on SIR and DEA. Similar reductions to the ones observed in the microcosm experiment (between 30 and 40%) of the microbial activities were observed in the planted pots exposed to CuO-NPs. In particular, SIR was inhibited by both 1 and 100 mg/kg CuO-NP treatments in the presence of wheat, and DEA was reduced when exposed to the highest concentration only. Thus, in this experiment, increased carbon resources provided by the plant did not clearly confer a higher resistance to CuO-NP exposure for the two heterotrophic soil microbial activities measured.

The reduction of SIR at 1 mg/kg was unexpected because this activity was not affected at the lowest concentrations in the absence of plants and was generally more resistant than DEA in the microcosm experiment. This result suggests that wheat presence modifies the bioavailability and toxicity of CuO-NPs as previously demonstrated (e.g., dissolution, adhesion on roots, uptake, Gao et al., 2018) or that the soil microbial community under the influence of the plant would be more sensitive to this pollutant than in the unplanted soil. More work is necessary to determine how plant exudates alter the aging of CuO-NPs in the rhizosphere and the sensitivity of the soil microbial community to this emerging contaminant.

NEA, a chemoautotrophic activity presenting a lower reliance on organic carbon than SIR and DEA, was not positively affected by plant presence and even decreased slightly in planted soils. The lack of stimulation of NEA in the presence of wheat can be explained by a strong competition for ammonia between nitrifiers and plants (Cantarel et al., 2015). This alteration of the N cycle in presence of the plant was also highlighted by a stronger correlation observed between NEA and DEA in the unplanted soils. Interestingly, the CuO-NP treatment did not significantly alter NEA in the pot experiment, indicating that the competition for N resources between nitrifiers and the plant did not increase their sensitivity to the contaminant. These results show that the effects of plant presence on CuO-NP toxicity vary according to the microbial activity and the type of plant-microorganism interaction involved (commensalism vs. competition).

In the context of the potential use of CuO-NPs in agro-chemical products, our results indicate that at low concentrations (1 mg/kg), CuO-NP soil application could lead to an increase in wheat biomass. In our study, these effects were due to a higher allocation of biomass to the roots than to the leaves, which increased the total plant biomass. The stimulatory effect of CuO-NPs on the root system could be explained by different mechanisms, such as the use of dissolved CuO-NPs as micronutrients by plants (as suggested by Dimkpa et al., 2013), the elimination of plant pathogens by CuO-NPs (Hajipour et al., 2012) or a plant stress response leading to a higher energy allocation to root growth to compensate the energy costs associated with CuO-NP detoxification (Potters et al., 2007). To determine the potential value of applying CuO-NP to wheat crops, future studies will need to determine the effects of CuO-NP exposures on the agroecosystems and their productivity (as grain production, mass and quality) and assess the long-term effects of the alteration of soil microbial activities on soil fertility.

## Conclusion

These two experiments show that CuO-NPs can have detrimental effects on soil microbial activities, but most effects occurred at the highest concentration tested (100 mg/kg). Similar to previous studies, we observed that the negative effects of CuO-NPs increase over time, indicating that short-term studies (hours, days) may underestimate the risks posed by these contaminants. The effects differed between the five soils studied, but all soils presented significant reductions in microbial activity. Our results indicated that the most impacted soil was a loam soil with a low OM content (LCSA), though more research is necessary to determine which biotic and abiotic characteristics are the main drivers of this soil sensitivity to CuO-NPs. Additionally, this work demonstrates that the presence of plants influences the microbial response to CuO-NP exposure but does not mitigate or compensate the effects. For example, large decreases in heterotrophic microbial activities were observed in planted soils, even at 1 mg/kg for SIR. Altogether, this study provides a clear demonstration of the necessity to assess the environmental impacts of nanomaterials under realistic experimental conditions to improve the risk assessment of these novel contaminants. Future studies in nanotoxicology need to include systematically low concentrations (μg/kg and low mg/kg range) and take into account soil biological complexity and physico-chemical diversity in their experimental designs to produce an integrative assessment useful for regulation.

## Author Contributions

MS, AAMC, AC, JM, and AR designed the experiments. MS, AAMC, JG, and AC conducted the experiments and performed the plant and microbial measurements. MS, AAMC, and AR wrote the paper and all the co-authors edited the manuscript.

### Conflict of Interest Statement

The authors declare that the research was conducted in the absence of any commercial or financial relationships that could be construed as a potential conflict of interest.
