# Supplementary material for: Negative Effects of Copper Oxide Nanoparticles on Carbon and Nitrogen Cycle Microbial Activities in Contrasting Agricultural Soils and in Presence of Plants
Source: Front Microbiol. 2018 Dec 13;9:3102. doi: 10.3389/fmicb.2018.03102 (PMC6301197; doi:10.3389/fmicb.2018.03102)
Supplement: Supplementary file 1 [file Table_1.DOCX]

**SUPPLEMENTARY INFORMATION**

**Negative Effects of Copper Oxide Nanoparticles on Carbon and Nitrogen Cycle Microbial Activities in Contrasting Agricultural Soils and in Presence of Plants**

**Marie Simonin^1,2,3^, Amélie A.M. Cantarel^1,2,3^, Armelle Crouzet^1,2,3^, Jonathan Gervaix^1,2,3^, Jean M.F. Martins^4^, Agnès Richaume^1,2,3^**

^1^Université de Lyon, Lyon, France

^2^Université Claude Bernard Lyon 1, Villeurbanne, France

^3^CNRS, UMR 5557, Microbial Ecology Centre, Université Lyon 1, Villeurbanne, France

^4^Université Grenoble Alpes, CNRS, IRD, IGE, Grenoble, France

*** Correspondence:**Marie Simonin
simonin.marie@gmail.com

**Keywords:** Metal-oxide nanomaterials, Agro-ecosystem, Microbial Ecotoxicology, Wheat, Nitrification, Denitrification, Soil respiration, Plant-Microorganism interactions


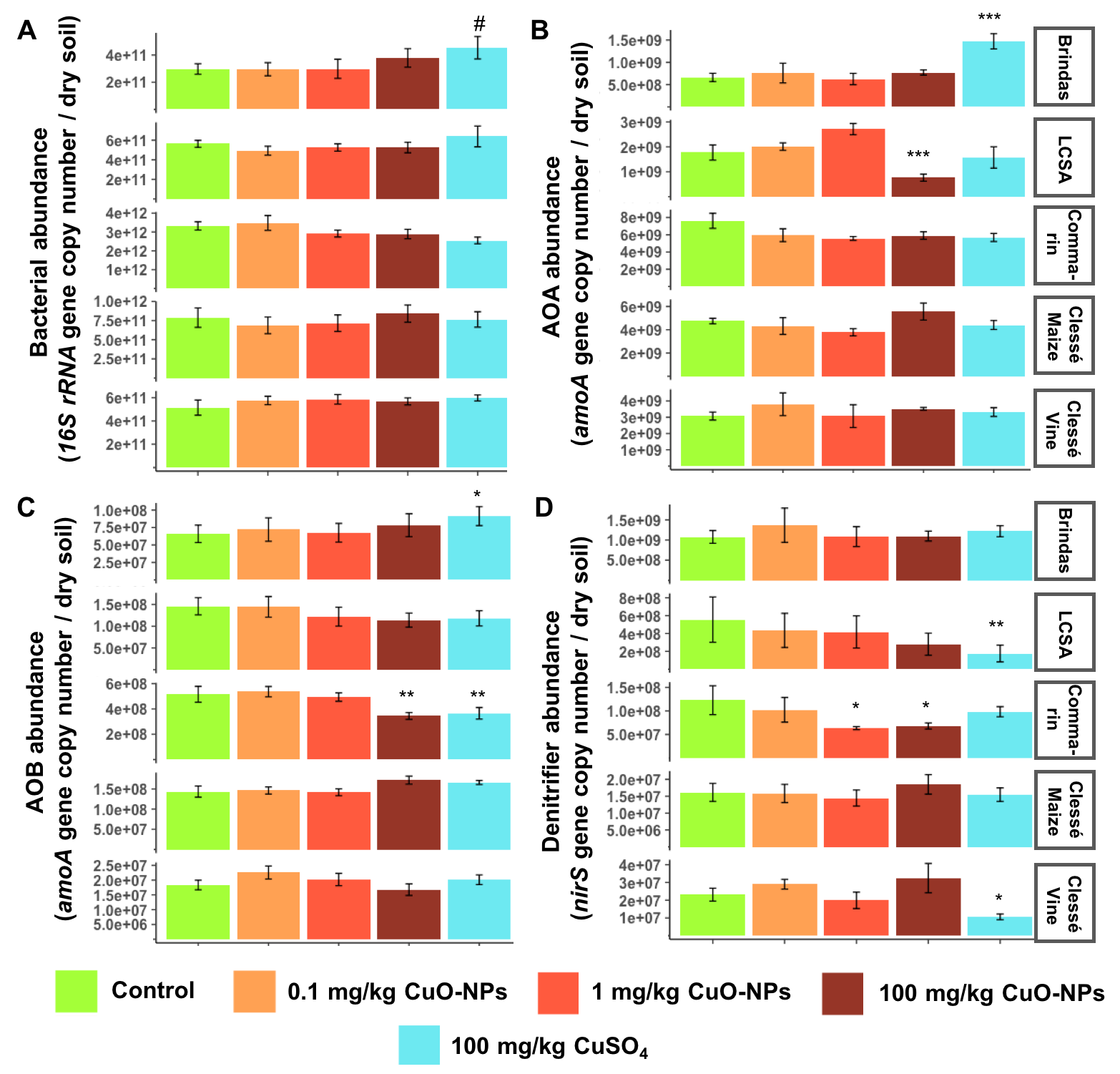


Figure S1: Effects of the treatments on microbial abundances: A) Bacterial abundance, B) AOA abundance, C) AOB abundance and D) Denitrifier abundance. The asterisks represent the significant effects of the treatments compared to the controls: #, *P*<0.1; *, *P*<0.05; **, *P*<0.01; ***, *P*<0.001. Note that the scale of the y-axes is different for each soil.
